# Re-imagining malaria – a platform for reflections to widen horizons in malaria control

**DOI:** 10.1186/s12936-015-0703-6

**Published:** 2015-04-24

**Authors:** Susanna Hausmann-Muela, Julian Eckl

**Affiliations:** Federal Department of Foreign Affairs FDFA, Swiss Agency for Development and Cooperation SDC, Freiburgstrasse 130, Bern, 3003 Switzerland; University of Hamburg, Hamburg, Germany

**Keywords:** Social determinants, Political economy, Social sciences, History, Malaria models and metaphors, Malaria control, Multisectoral approaches, Transdisciplinary research

## Abstract

Ongoing political-economic discussions that take stock of social and societal determinants of health present an opportunity for productive dialogue on why current approaches to malaria control and elimination need to be broadened, and how this may be accomplished. They invite us, for example, to look beyond malaria as a disease, to appreciate the experiences of malaria-afflicted populations, to transcend techno-centric approaches, to investigate social conflicts around malaria, to give voice to the communities engaged in bottom-up approaches, and to revisit lessons learned in the past.

While contributions from all disciplines are invited to this discussion, social scientists are particularly encouraged to participate. They have struggled in the past to find an appropriate platform within the malaria community that provides them the opportunity to address researchers from other disciplines, malaria practitioners, and policy makers. The *Malaria Journal*’s new thematic series on ‘re-imagining malaria’ offers them this opportunity. The goal of the series is to encourage transdisciplinary thinking, to stimulate discussion, to promote constructive criticism, and to gather overlooked experiences that help to reflect on implicit assumptions. Overall it aims at widening horizons in malaria control.

## The current image of malaria and its limits

Roll Back Malaria’s (RBM) nearly finalized second-generation *Global Malaria Action Plan* for the years 2016–2030 calls for a widening of antimalarial engagement to sectors beyond the health sector and for an active involvement of governments, civil society, and private business [1]. To make such widened approaches workable rather than just fashionable, it is essential to recapitulate what has (and has not) been achieved in malaria control this far and to reflect on the implicit assumptions that inform contemporary anti-malarial strategies.

Despite recent biomedical-technological advances and increased financial commitments, malaria remains a pressing global health problem and the parasite reveals its endurance through resistance and resilience to current approaches. In light of these ongoing challenges, it seems prudent to complement the efforts to increase the quantity and quality of technological interventions with alternative approaches to malaria control, for example, by drawing on underrepresented research traditions such as the social sciences or by reconsidering local perspectives.

Revisiting the history of malaria control is also instructive. While some perspectives on malaria, such as the miasma theory, are no longer considered plausible, the historical record equally contains examples of insightful observations that speak to a broader malaria agenda. To give one example, in his book *Christ Stopped at Eboli*, Italian artist, physician and political dissident Carlo Levi provides an evocative description of malaria. He experienced the disease first-hand while practicing medicine in a remote village of Lucania, Italy, to which he had been exiled during the Fascist regime in the 1930s:

*“In this region malaria is a scourge of truly alarming proportions; it spares no one and when it is not properly cared for it can last a lifetime. Productive capacity is lowered, the race is weakened, the savings of the poor are devoured; the result is a poverty so dismal and abject that it amounts to slavery without hope of emancipation. Malaria arises from the impoverishment of the deforested clayey land, from neglected water, and inefficient tilling of the soil; in its turn it generates in a vicious circle the poverty of the peasants. Public works on a large scale are necessary to uproot it”* [2].

Levi’s description evokes an image of poverty and desolation. The solution to malaria, he argues, is economic development of the region, an opinion that he shared with other contemporary Italian scientists, including the renowned malariologist Angelo Celli.

This stands in stark contrast to today’s prevailing image of malaria. Presently, it is framed less as a disease of underdevelopment and social deprivation but more as a biotechnological problem related to disrupting the parasite’s life cycle and warranting innovative technology to ‘fight’ the parasite or its mosquito vector. From this perspective, malaria can be ‘combated’ independent of social context by relying on a powerful tool-box of insecticides, drugs, bed nets, and soon vaccines. While the framing of the malaria problem in terms of military metaphors makes the development and application of new or improved tools appear as the logical answer that will win the ‘war’ on malaria, other dimensions of the malaria problem drop out of the picture. As Donald Schön argued decades ago, *“the framing of problems often depends upon metaphors underlying the stories which generate problem setting and set the direction of problem solving”* [3]. As with any complexity-reducing device, metaphors become troublesome when the model that they convey is taken as a definite account of a complex problem (in this case malaria) – leaving no room for other interpretations. Models can become ‘realities’ which curtail innovative thinking and historical analysis can unveil such processes.

To be sure, the massive mobilization of affordable prevention and treatment measures, which target the parasite and/or its vector, has had an important impact. According to the *World Malaria Report 2014*, the estimated number of people dying from malaria has fallen dramatically since 2000 and estimated malaria cases are also steadily declining. Between 2000 and 2013, the estimated malaria mortality rate decreased by 47% worldwide and by 54% in the WHO African Region, where about 90% of estimated malaria deaths occur [4]. On the other hand, it is difficult to evaluate the relative importance of technological interventions against the backdrop of socio-economic development, and increasing scientific evidence supports the role of socio-economic development as a sustainable intervention in the decline of the malaria burden [5].

While the current image of malaria helps to envision the technical-biomedical dimension of the malaria problem it simultaneously acts as a constraining ‘blinker’ that obscures the social determinants of health as well as the political economy of actually providing technological-biomedical solutions in a given context. A biotechnical gaze has deflected our vision from other, complex realities of what malaria means to different people – in different places and at different times. Political, economic, cultural, social and environmental determinants all define the specific circumstances of malaria and shape people’s lived experiences of the illness.

Already 40 years ago, the Special Programme for Research and Training in Tropical Diseases (TDR) recognized a need to address the control of infectious diseases from a development perspective. TDR programs promoted research in support of designing strategies for health improvement based on social, economic and behavioral realities [6]. However, over the past decades, the focus on innovation for new tools against malaria overshadowed the need for a broader scope, and despite a long-track record of taking ‘the social’ into account, malaria research has not received the attention in transdisciplinary research that it would deserve.

Malaria is a complex disease that requires holistic, systemic and politically engaged responses.

Moreover, successes always run the risk of being followed by stagnation or even by reversion. Drawing either on experience or on systems theory, one should furthermore expect that antimalarial interventions will inevitably lead to unintended consequences. In light of these considerations, the call for ‘re-imagining malaria’ should not be interpreted as a one-time activity but as an open-ended process in the course of which research designs that can account for the unexpected might prove particularly relevant.

## The specific background of this initiative

A workshop titled “Re-imagining malaria: looking into, behind and beyond current priorities”, convened in September 2014 by the London School of Hygiene and Tropical Medicine, brought together social scientists from eleven institutions who work in malaria research and control. During the workshop, participants discussed the tendency to represent the disease in a reductionist fashion that runs the risk of overlooking important dimensions of malaria control and in so doing can lead anti-malarial efforts astray [7].

Participants drew attention to the important role of several factors beyond technology including: general health infrastructures (staff training, facilities, salaries, material and disposable supplies, equipment), the built environment (housing, urbanization, sanitation), and political-economic factors influencing local communities that need to be taken under consideration (poverty, nutrition, migration, capital flows, accountability, political stability, and trust between civic actors). Re-structuring the way in which malaria is addressed beyond the health sector requires not only multisectoral, but inter- and transdisciplinary approaches to both development and disease control. Malaria elimination in Italy relied heavily on just such broad based disease control measures that entailed serious thought given to development and social issues that had health benefits beyond malaria.

At the workshop, concerns were raised that despite the fact that malaria programmes are implemented in completely different contexts, a majority of funding for malaria is invested in standardized technological solutions, such as bed nets, rapid diagnostic tests, and insecticide residual spraying. Far too little attention is paid to implementation challenges and conflicts between local needs and universal solutions. Accessibility, acceptability, and integration of tools into local health care systems all depend on the support of local populations and experienced public health staff, yet their perspectives are commonly overlooked when health campaigns are planned. Local perceptions of risk, divergent explanatory models of cause, and context-dependent responses to malaria as one of many types of fever that people face are little considered as are the (in-)direct and opportunity costs of treating malaria, including impacts on household economies. Participants felt that both the political economy and the lived experiences of malaria needed to be paid greater credence and that the many like-minded but dispersed people within the malaria community needed a space for discussing and disseminating their insights and visions. The *Malaria Journal’s* thematic series on ‘re-imagining malaria’ seeks to provide such a platform and welcomes contributions from all disciplines.

## Why re-imagining malaria now – and how to proceed from here?

The year 2015 offers the perfect setting for initiating inter- and transdisciplinary dialogue around re-imagining malaria. After a long political process, 2015 marks the transition from the Millennium Development Goals to the Sustainable Development Goals, explicitly addressing the social, cultural, economic, environmental and political determinants of health. In the specific case of malaria, two high-level global strategic documents will shape visions and goals as well as determine targets and indicators for malaria control and elimination over the next 15 years: RBM’s *Global Malaria Action Plan 2* and the World Health Organization’s *Global Malaria Technical Strategy*. Both have been crafted in a synchronous and collaborative process, and are expected to promote a vision of malaria control and elimination that calls for more integrated approaches. Each speaks to the importance of fostering country ownership and leadership, promoting the participation of communities, and the adoption of a development perspective that extends its focus beyond the health sector.

The goal of the thematic series ‘Re-imagining malaria’ is to promote dialogue on how this may be accomplished best, to invite constructive criticism of existing and future programmes, as well as to foster innovative thinking. The thematic series welcomes articles, reviews, and commentaries that lead us to think about both the ‘whys’ and ‘hows’ of a broader understanding of malaria and its control.
